# Associations of serum carotenoids with the severity of sunburn and the risk of cancer: A cross-sectional analysis of 1999–2018 NHANES data

**DOI:** 10.3389/fnut.2022.1051351

**Published:** 2022-12-20

**Authors:** Bin Cheng, Xixin Wu, Ruina Li, Jiayuan Tu, Sixian Lin, Xiangda Zhang, Xiaoqiao Mo, Tian Xie

**Affiliations:** ^1^Department of Burns and Plastic Surgery, Union Shenzhen Hospital, Huazhong University of Science and Technology, Shenzhen, Guangdong, China; ^2^Health Science Center, Shenzhen University, Shenzhen, China; ^3^Department of Clinical Pharmacy, Union Shenzhen Hospital, Huazhong University of Science and Technology, Shenzhen, Guangdong, China; ^4^School of Nursing and Public Health, Yangzhou University, Yangzhou, China; ^5^Department of Public Health and Preventive Medicine, Monash University, Melbourne, VIC, Australia; ^6^Department of Operating Room, Xinhua Hospital, Shanghai Jiao Tong University School of Medicine, Shanghai, China; ^7^Department of General Surgery, Shanghai Ninth People’s Hospital, Shanghai Jiao Tong University School of Medicine, Shanghai, China

**Keywords:** sunburn, carotenoids, cancer, melanoma, skin cancer

## Abstract

**Background:**

Sunburn is a common problem for outdoor workers and casual outdoor walkers. Carotenoids are important elements in normal function of skin tissue and skin metabolism and are critical in the development of some cancers. However, the possible relationships between sunburn sensitivity, carotenoids and the risk of cancers remain unknown.

**Objectives:**

To explore the associations of serum carotenoids with sunburn severity and the risk of cancers.

**Methods:**

A cross-sectional study from the National Health and Nutrition Examination Survey from 1999 to 2018 were conducted. The relationship between sunburn and serum carotenoids, cancers were investigated by unconditional or ordinal logistic regression. Mediation analysis was used to explore the effect of carotenoids on the relationship between sunburn and cancers.

**Results:**

A total of 25,440 US adults from 1999 to 2018 were enrolled in this study. There were significant differences in sex, race and natural hair color between the sunburn and non-sunburn people. The severity of sunburn was significantly associated with serum *trans*-β-carotene, *cis*-β-carotene, combined lutein, and vitamin A. The odds ratios of severe reactions were 5.065 (95% CI: 2.266–11.318) in melanoma patients, 5.776 (95% CI: 3.362–9.922) in non-melanoma patients, and 1.880 (95% CI: 1.484–2.380) in non-skin cancers patients. Additionally, serum carotenoids were partially attributable to the effect of sunburn on skin and non-skin cancers.

**Conclusion:**

Sunburn severity was associated with cancers, and severer sunburn was related with higher risk of cancers. Serum carotenoids were also associated with sunburn severity. Moreover, the relationship between sunburn and cancers was mediated by some serum carotenoids.

## Introduction

Skin is the body’s largest organ and acts as a physical barrier protecting against a variety of environmental risk factors, such as microbes, injuries, toxins and ultraviolet radiation (1). Skin can also serve as a biological guard to decrease body water loss, regulate body temperature and synthesize vitamin D. The main mechanism of sunburn is due to ultraviolet (UV) type A and B. Most of UVA and a small amount of UVB radiation can reach the Earth’s surface and come into contact with the skin due to blockage by ozone (2). UV-A radiation can invade the deepest skin tissues, causing oxidative damage and skin aging, but it does not cause direct DNA damage. UV-B radiation cannot penetrate the skin tissues and only remains in the dermis layer. UV-B can cause oxidative stress and an inflammatory response (3). In addition, UV-B may act on NDA, causing pigmentation effects, sunburn and malignant skin tumors (4). Sunlight brings many problems to outdoor walkers and susceptible people, such as sunburn, skin condition or malignancies. Sunlight some pose high risks to human health and lifestyles with a very large economic burden.

Carotenoids are fat-soluble natural pigments widely distributed in nature. They are widely found in colorful fruits and vegetables that are commonly found in the daily diet, such as carrots, tomatoes, corn, peppers, and some marine algae (5). Serum carotenoids cannot be synthesized in the human body, and food is the only way to obtain carotenoids. Carotenoids have many potential functions, such as antioxidation, antitumor activity, immune function enhancement, vision protection, skin protection and participation in reproduction and development (6). Carotenoids can be divided into two groups, carotene and lutein. Carotenes include α-carotene, β-carotene, and lycopene. Lutein includes zeaxanthin, α-cryptoxanthin, β-cryptoxanthin, astaxanthin, fucoxanthin, and so on. Carotenoids have attracted the attention of the scientific community and the public in recent years because of their potential health-promoting effects.

A large number of studies have shown that carotenoids have a photoprotective effect; they can not only directly absorb ultraviolet rays from the sun but also have antioxidant effects, removing reactive oxygen species and oxygen free radicals in the skin, reducing skin photodamage and preventing sunburn (7–9). Studies have shown that serum carotenoids have an inhibitory effect on the occurrence and development of various malignant tumors. However, the relationships among serum carotenoids, sunburn severity and skin malignancy remain unclear. The purpose of this study was to investigate the association of serum carotenoids and vitamin A with sunburn severity and skin cancers.

## Materials and methods

### Design and population

**FIGURE 1:**
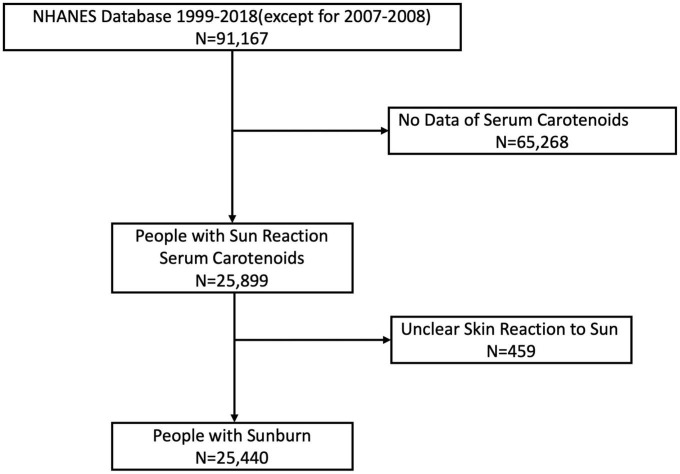
Flowchart of study participants.

The National Health and Nutrition Examination Survey (NHANES) is a research program designed to assess the health and nutrition status of adults and children in the United States. Data were obtained via personal structured interviews at home, health examinations at a mobile examination center, and specimen analyses in the laboratory (10). All participants provided written consent. The protocol for NHANES was approved by the National Center for Health Statistics Research Ethics Review Board. We extracted data from NHANES 1999 to 2018, with key information on skin reaction to sun for half an hour after months of non-sun exposure and serum carotenoids, except for the data of 2007–2008 NHANES, which lacks skin reaction information. We excluded participants who lacked exact information on skin reactions, resulting in 25,440 participants in the final analysis (Figure 1). The degrees of sunburn were divided into four groups: none (nothing would happen in half an hour), mild (turning darker without a sunburn), moderate (mildly burned with some tanning) or severe (severe sunburn for a few days with peeling or getting a severe sunburn with blisters). Data described in the manuscript, code book, and analytic code will be made publicly and freely available without restriction at https://www.cdc.gov/nchs/nhanes/index.htm.

### Measurement of carotenoids

The serum carotenoids α-cryptoxanthin, β-cryptoxanthin, α-carotene, *trans*-β-carotene, *cis*-β-carotene, total β-carotene, *trans*-lycopene, *cis*-lycopene, total lycopene, phytoene, phytofluene, zeaxanthin, lutein, *cis*-lutein/zeaxanthin, combined lutein/zeaxanthin, retinyl palmitate, retinyl stearate, and vitamin A were measured using high-performance liquid chromatography (HPLC) or a comparable HPLC method over different durations according to the corresponding codebooks in detail. Some duration data from NHANES were then converted by a regression method to equivalent carotenoid measurements from the HPLC method. The laboratory procedures and quality control methods for serum carotenoid measurements are described in detail elsewhere. Further details of these measurements were documented in the NHANES Laboratory Medical Technologists Procedures Manual.

### Assessment of covariates

Standardized questionnaires were used to obtain information on demographic characteristics, smoking status, alcohol consumption, and liver and kidney diseases. Those who smoked < 100 cigarettes in their lifetime were classified as never smokers, those who smoked > 100 cigarettes in their lifetime were classified as current smokers, and those who smoked > 100 cigarettes and had quit were classified as former smokers. Alcohol consumption status was classified as non-drinker, drinker (drinking now), or former drinker (drank but quit). BMI was calculated as weight/height^2^ (kg/m^2^). The liver and kidney disease statuses were determined according to self-report questionnaires. Both melanoma and non-melanoma were included as skin tumors.

### Statistical analysis

All analyses incorporated sample weights and primary sampling units to produce accurate national estimates. Sample characteristics are reported as the means (standard deviations) for normally distributed continuous variables and numbers (percentages) for categorical variables. The difference between the sunburn and non-sunburn groups was analyzed by Student’s *t*-test was used for continuous variables with a normal distribution, Kruskal-Wallis test was used for non-normally distributed continuous variables, and χ^2^ test/Fisher’s exact test was used for categorical variables. The association of serum carotenoids with the severity of sunburn was analyzed by using multilevel ordinal logistic regression, adjusting for BMI, age, race and sex (Model 1) or BMI, age, race, sex, smoking status and natural hair color (Model 2). The association of the severity of sunburn with skin cancers and non-skin cancer was analyzed by binary logistic regression models and odds ratios (ORs with 95% confidence intervals). The moderation model was selected to test whether carotenoids moderated the relationship between sunburn and skin tumors. The 95% confidence interval of the bootstrapping method did not include 0, and there was no significant difference in the mediation model. All analyses were performed using R software. Two-sided *p* < 0.05 was considered statistically significant.

## Results

A total of 14,644 participants with sunburn injuries and 10,796 without sunburn injuries were enrolled in our study (Figure 1). There was no significant difference in age between the two groups. The proportion of females was higher in the sunburn group than in the non-sunburn group. The natural hair colors of sunburn participants were mostly brown and black (60.5 vs. 23.0%), while the proportion in the non-sunburn group was 48.9 vs. 44.8%. Most sunburn participants were non-Hispanic white (52.7%), while most non-sunburn participants were non-Hispanic black and white (34.5 and 25.9%). Other sociodemographic and laboratory data of sunburn and non-sunburn participants are summarized in Table 1.

**TABLE 1:** Sociodemographic, health characteristics, and serum carotenoids of the sample of sunburn and non-sunburn in the United States, National Health and Nutrition Examination Survey 1999–2018.

	Non-sunburn (*N* = 10,796)	Sunburn (*N* = 14,644)	*P*- value
Age (years)	38.5 (11.5)	38.5 (11.2)	0.977
**Gender**			<0.001
Female	5,217 (48.3%)	8,077 (55.2%)	
Male	5,579 (51.7%)	6,567 (44.8%)	
Weight (kg)	82.5 (22.4)	81.7 (21.8)	0.006
Height (cm)	168 (10.1)	168 (9.98)	0.702
BMI (kg/m^2^)	29.1 (7.10)	28.8 (7.08)	0.005
**Hair color**	2,707	3,886	<0.001
Black	1,213 (44.8%)	894 (23.0%)	
Blonde	139 (5.1%)	496 (12.8%)	
Brown	1,324 (48.9%)	2,351 (60.5%)	
Other	6 (0.2%)	15 (0.4%)	
Red	25 (0.9%)	130 (3.3%)	
**Race**			<0.001
Mexican American	2,171 (20.1%)	2,439 (16.7%)	
Non-Hispanic Black	3,725 (34.5%)	1,780 (12.2%)	
Non-Hispanic White	2,794 (25.9%)	7,715 (52.7%)	
Other	2,106 (19.5%)	2,710 (18.5%)	
**Smoking**	10,785	14,639	<0.001
Former	1,640 (15.2%)	2,747 (18.8%)	
Never	6,443 (59.7%)	8,205 (56.0%)	
Current	2,702 (25.1%)	3,687 (25.2%)	
**Drinking**	6,368	9,581	<0.001
Drinker	2,861 (44.9%)	4,574 (47.7%)	
Former	305 (4.8%)	433 (4.5%)	
Non-drinker	3,202 (50.3%)	4,575 (47.8%)	
**Liver diseases:**	10,786	14,625	<0.001
No	10,525 (97.6%)	14,087 (96.3%)	
Yes	261 (2.4%)	539 (3.7%)	
**Kidney diseases**	10,781	14,627	0.214
No	10,596 (98.3%)	14,345 (98.1%)	
Yes	185 (1.7%)	282 (1.9%)	
α-Cryptoxanthin (μg/dL)	2.76 (1.27)	2.69 (1.30)	0.164
Total β-carotene (μg/dL)	15.8 (17.5)	16.5 (20.2)	0.341
*Cis*-lycopene (μg/dL)	20.5 (9.94)	20.7 (9.35)	0.600
*Cis*-lutein (μg/dL)	1.47 (0.74)	1.46 (0.86)	0.707
Total lycopene (μg/dL)	43.1 (20.0)	43.5 (19.0)	0.582
Lutein (μg/dL)	10.1 (5.06)	10.1 (4.90)	0.874
Phytofluene (μg/dL)	5.16 (3.10)	5.35 (3.29)	0.130
Phytoene (μg/dL)	4.09 (2.90)	4.15 (4.20)	0.699
Zeaxanthin (μg/dL)	4.12 (2.05)	4.08 (1.96)	0.548
α-Carotene (μg/dL)	4.02 (5.97)	4.30 (5.34)	0.021
*Trans*-β-carotene (μg/dL)	15.4 (16.8)	16.8 (18.4)	<0.001
*Cis*-β-carotene (μg/dL)	1.03 (1.06)	1.10 (1.11)	0.004
β-Cryptoxanthin (μg/dL)	10.8 (9.28)	10.2 (8.61)	0.007
Combined lutein (μg/dL)	16.3 (8.16)	15.9 (8.32)	0.038
*Trans*-lycopene (μg/dL)	23.6 (11.0)	24.3 (10.8)	0.002
Retinyl palmitate (μg/dL)	1.92 (1.67)	2.11 (2.07)	<0.001
Retinyl stearate (μg/dL)	0.44 (0.38)	0.47 (0.48)	0.002
Vitamin A (μg/dL)	54.4 (16.2)	57.2 (16.6)	<0.001
**Non-skin cancers**	10,751	14,431	<0.001
No	10,512 (97.8%)	13,964 (96.8%)	
Yes	239 (2.2%)	467 (3.2%)	
**Skin cancers**			<0.001
None	10,746 (99.5%)	14,481 (98.9%)	
Skin cancers (non-melanoma)	39 (0.36%)	124 (0.85%)	
Melanoma	11 (0.10%)	39 (0.27%)	

To explore the relationship between serum carotenoids and the degrees of sunburn, univariate and multivariate ordinal logistic regression (OLR) were used to create a proportional odds model. Multivariate odds ratios were adjusted for BMI, age, race and sex (Model 1) or BMI, age, race, sex, smoking status and natural hair color (Model 2). Combined lutein and vitamin A were associated with the degrees of sunburn after adjusting for Model 1 or Model 2 (Table 2). Notably, *trans*-β-carotene and *cis*-β-carotene were associated with sunburn after adjustment, while the raw odds ratio showed no significant difference, this may because of the negative impact of BMI on the relationship between β-carotene and sunburn (*r* = −0.18, *p* = 0.005). The overall results of our ordinal logistic regression model for carotenoids in the sunburn population are summarized in Table 2.

**TABLE 2:** The association between the severity of sunburn and serum carotenoids.

					Multivariate analysis							
		
	Univariate analysis				Model 1				Model 2			
			
	Odds ratio	Lower	Upper	*p*	Odds ratio	Lower	Upper	*p*	Odds ratio	Lower	Upper	*p*
α-Cryptoxanthin	0.817	0.759	0.878	<0.001	0.976	0.899	1.059	0.559	0.978	0.898	1.064	0.604
Total β-carotene	0.998	0.994	1	0.455	0.997	0.9914	1.002	0.223	0.997	0.9912	1.002	0.242
*Cis*-lycopene	1	0.99	1.01	0.942	0.997	0.9864	1.008	0.584	0.997	0.9859	1.007	0.537
*Cis*-lutein	0.795	0.704	0.895	<0.001	0.973	0.8558	1.099	0.667	0.958	0.845	1.084	0.496
Total lycopene	1	0.996	1.01	0.631	1	0.9948	1.005	0.992	1	0.9944	1.005	0.904
Lutein	0.964	0.946	0.983	<0.001	0.98	0.9595	1.001	0.065	0.98	0.959	1.002	0.079
Phytofluene	1	0.976	1.03	0.833	1.002	0.973	1.033	0.871	1	0.9706	1.031	0.976
Phytoene	0.997	0.975	1.02	0.743	1	0.9774	1.022	0.983	1.002	0.979	1.024	0.833
Zeaxanthin	0.892	0.85	0.936	<0.001	0.975	0.9245	1.028	0.355	0.979	0.9266	1.034	0.439
α-Carotene	1	0.993	1.01	0.554	0.994	0.984	1.005	0.267	0.989	0.975	1.003	0.124
*Trans*-β-carotene	1	0.997	1	0.846	0.996	0.993	0.999	0.017	0.996	0.992	1	0.043
*Cis*-β-carotene	0.976	0.931	1.02	0.293	0.93	0.882	0.98	0.007	0.932	0.872	0.993	0.032
β-Cryptoxanthin	0.978	0.971	0.984	<0.001	0.997	0.989	1.004	0.341	0.999	0.99	1.008	0.822
Combined lutein	0.981	0.975	0.987	<0.001	0.99	0.983	0.996	0.002	0.99	0.981	0.999	0.035
*Trans*-lycopene	1	0.996	1.01	0.798	0.999	0.994	1.004	0.686	0.995	0.989	1.002	0.151
Retinyl palmitate	1.01	0.984	1.04	0.471	0.994	0.967	1.021	0.676	1.009	0.976	1.042	0.599
Retinyl stearate	1.09	0.973	1.21	0.144	1.023	0.907	1.152	0.713	1.112	0.969	1.278	0.13
Vitamin A	1.01	1.01	1.01	<0.001	1.004	1	1.007	0.048	1.006	1.001	1.01	0.012

Model 1: Adjusted for BMI, age, race, and gender. Model 2: Adjusted for BMI, age, race, gender, smoking status, and natural hair.

Next, unconditional or ordinal logistic regression was used to analyze the relationships between cancers (both cutaneous and non-cutaneous) and the degrees of sunburn. Compared to the people who had no skin reaction to the sun after non-exposure, the more severe the people reacted to the sun, the higher the risk of non-cutaneous reactions, with ORs of 1.244 (95% CI: 1.017–1.523) for mild exposure, 1.526 (95% CI: 1.262–1.845) for moderate exposure and 1.880 (95% CI: 1.484–2.380) for severe exposure. Moreover, the same phenomenon was seen regarding skin cancers in the moderate and severe reaction groups, with ORs of 2.827 (95% CI: 1.983–4.031) and 4.629 (95% CI: 3.143–6.818), respectively (Table 3). Specifically, the ORs of moderate and severe reactions were 3.052 (95% CI: 1.452–6.418) and 5.065 (95% CI: 2.266–11.318), respectively, in melanoma patients and 3.764 (95% CI: 2.291–6.183) and 5.776 (95% CI: 3.362–9.922), respectively, in non-melanoma patients, while only severe reactions, with an OR of 2.679 (95% CI: 1.214–5.910), were seen in people with unknown kinds of skin cancers (Table 3).

**TABLE 3:** The association between the severity of sunburn and cancers.

	95% confidence interval			
	
Predictor	Odds ratio	Lower	Upper	*p*
Skin cancers				
Mild–No	1.058	0.669	1.673	0.811
Moderate–No	2.827	1.983	4.031	<0.001
Severe–No	4.629	3.143	6.818	<0.001
Non-skin cancers				
Mild–No	1.244	1.017	1.523	0.034
Moderate–No	1.526	1.262	1.845	<0.001
Severe–No	1.880	1.484	2.380	<0.001
Skin cancers with categories				
Melanoma–No				
Mild–No	1.161	0.450	2.995	0.758
Moderate–Noe	3.052	1.452	6.418	0.003
Severe–No	5.065	2.266	11.318	<0.001
Skin (non-melanoma) –No				
Mild–No	1.110	0.571	2.158	0.759
Moderate–No	3.764	2.291	6.183	<0.001
Severe–No	5.776	3.362	9.922	<0.001
Skin (don’t know what kind)–No				
Mild–No	0.912	0.390	2.132	0.831
Moderate–No	1.325	0.627	2.803	0.461
Severe–No	2.679	1.214	5.910	0.015

**FIGURE 2:**
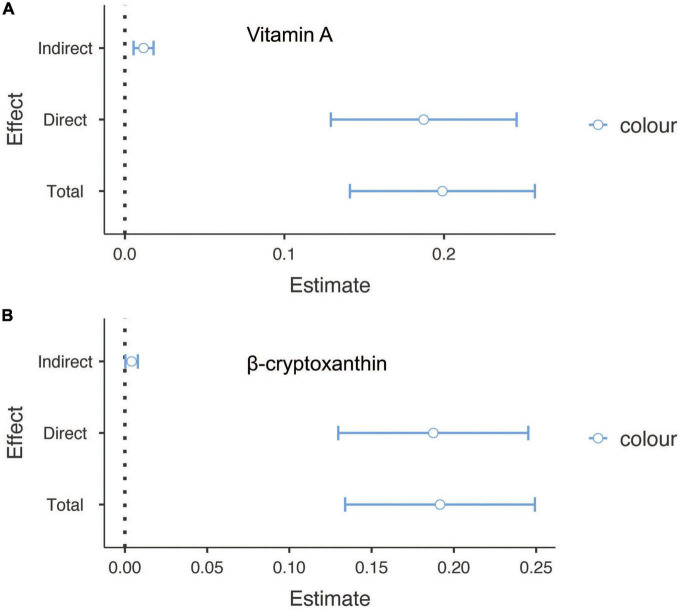
The mediation analysis for serum carotenoids in sunburn and skin cancers. **(A)** The mediation effect of vitamin A in sunburn and skin cancers. **(B)** The mediation effect of β-cryptoxanthin in sunburn and skin cancers.

**FIGURE 3:**
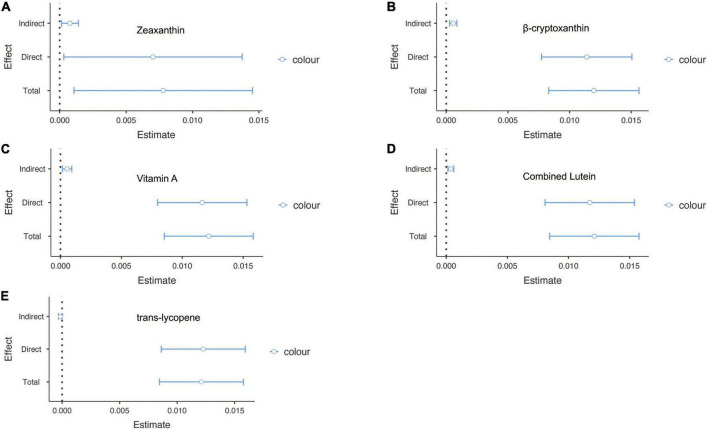
The mediation analysis for serum carotenoids in sunburn and non-skin cancers. **(A)** The mediation effect of zeaxanthin in sunburn and skin cancers. **(B)** The mediation effect of β-cryptoxanthin in sunburn and skin cancers. **(C)** The mediation effect of vitamin A in sunburn and skin cancers. **(D)** The mediation effect of combined lutein in sunburn and skin cancers. **(E)** The mediation effect of *trans*-lycopene in sunburn and skin cancers.

Finally, to confirm whether the effect of sunburn on cancer was partially mediated by carotenoids, causal mediation analysis was conducted. Among people with skin cancers, vitamin A and β-cryptoxanthin were attributable to skin cancers with mediated proportions of 5.89 and 2.14, respectively (*p* < 0.05) (Figure 2). In addition, zeaxanthin, β-cryptoxanthin, vitamin A, combined lutein and *trans*-lycopene accounted for partial mediation of the effect of sunburn on non-skin cancers, with proportions of 9.88, 4.79, 4.45, 3.11, and 1.32, respectively (*p* < 0.05) (Figure 3).

## Discussion

This study is the first to assess the association of sunburn severity with serum carotenoids and the risk of cancer risk in a nationally representative sample of sunburn patients in the United States. Our results demonstrated that the severity of sunburn after non-exposure was associated with serum carotenoids and the risks of both skin and non-skin cancers. In addition, serum carotenoids contributed to the mediation effect of sunburn on cancers. Sunburn participants were more likely to be female, have brown natural hair color and be non-Hispanic white. Higher serum *trans*-β-carotene, *cis*-β-carotene, and combined lutein concentrations were associated with a lower degree of sunburn, while higher serum vitamin A levels were associated with a higher degree of sunburn.

As a group of hydrophobic antioxidants, serum carotenoids can be scavenged by oxidation, reduction, hydrogen abstraction and addition. By quenching singlet oxygen and neutralizing oxygen radicals, carotenoids may inhibit oxidative stress and inflammatory responses (11). In a double-blind, placebo, randomized controlled 10-week study, participants in the 4 mg-daily astaxanthin supplementation group lost less skin moisture and had significantly better skin texture in non-UV-irradiated areas than individuals in the placebo group (12). In another 5-week double-blind, placebo-controlled study, 9 mg-daily lutein supplements showed that lutein could reduce oxidative stress and inhibit UV-induced skin dullness and aging (13). A meta-analysis of seven experiments showed that β-carotene supplementation was effective in preventing sunburn and found that dietary supplementation with β-carotene was a time-dependent way to provide sun protection (14). However, other studies have shown that serum carotenoids may have pro-oxidative effects at high concentrations, which may exert harmful effects on the human body (15). However, recent studies have shown that in addition to driving the antitumor immune responses of dendritic cells, reactive oxygen species are crucial for damaging cancer cells in the contexts of chemotherapy and multidrug resistance (16). Our results showed that lower serum carotenoid concentrations were associated with higher levels of sunburn, which may be associated with antioxidant activity.

The development of skin cancer is directly related to long-term exposure to ultraviolet radiation (17). Studies have shown that epidermal cells may activate phosphoinositide-3-kinase/protein kinase B (PI3K/Akt) and mitogen-activated protein kinase (MAPK) signaling pathways under UV stimulation, which phosphorylates downstream substrates and ultimately leads to skin cancer (18). A systematic meta-analysis of 24 observational studies involving 16,180 melanoma cases revealed that areas that frequently experienced sunburn were at higher risk than other areas of the body for the development of melanoma (19). Our results also showed that both skin and non-skin cancers were more commonly seen in severe sunburn patients. Compared with people with no sunburn after sun exposure, severe sunburn participants had a higher risk of non-skin cancers, skin melanoma, non-melanoma skin cancers and non-skin cancer, with odds ratios of 1.880 (95% CI: 1.484–2.380), 5.065 (95% CI: 2.266–11.318), and 5.776 (95% CI: 3.362–9.922), respectively.

Serum carotenoids can not only reduce oxidative stress but also enhance the proliferation of CD4+T cells and the activity of natural killer cells and play a certain role in inhibiting the occurrence and development of malignant tumors by regulating some cell signal transduction pathways (20). Sheng et al. showed that serum carotenoids may affect the MAPK and PI3K/Akt signaling pathways of zeaxanthin by increasing cycle-related proteins P21 and P27 and regulating the levels of CD44 and CD105 on the epidermal membrane, thus inducing apoptosis of human gastric cancer cells and inhibiting the proliferation of melanoma cells (18, 21). *In vitro* cell culture experiments also showed that zeaxanthin and lutein were cytotoxic to human colorectal adenocarcinoma cells and could inhibit their growth (22). Moreover, clinical controlled experiments showed that the intakes of zeaxanthin and lutein were negatively correlated with the risk of colorectal cancer (*OR* = 0.25, 95% *CI* = 0.18, 0.36) (23). A case-control study from South Korea showed that higher lycopene intake was inversely associated with gastric cancer risk in men (OR 0.60, 95% CI 0.42, 0.85) and women (OR 0.54, 95% CI 0.30, 0.96) (24). However, the relationship between serum vitamin A and malignancy has not been completely consistent. In a randomized controlled trial of 75,170 participants, the adjusted hazard ratio between higher vitamin A intake and squamous cell carcinoma was 0.83 (95% CI, 0.75–0.93) (25). An Italian case-control trial reported an adjusted odds ratio of 0.57 (95% CI 0.39, 0.83) between retinol intake and skin malignant melanoma, which indicated a significant negative association between retinol intake and skin malignant melanoma (26). However, a case-control study showed that a higher serum retinol concentration may be significantly positively associated with prostate cancer risk (OR: 1.74; 95% CI: 1.14, 2.68) (27). Other studies also found a significant negative correlation between serum retinol concentration and the risk of gastrointestinal malignancy (OR: 0.79; 95% CI 0.69, 0.91), and when serum vitamin A concentration was higher than 68.2 μg/dL, there was a significant positive correlation between serum retinol concentration and the risk of non-digestive malignancy (OR: 1.65; 95% CI, 1.12, 2.44) (28). In our study, the mediation effect of the effects of serum carotenoids and vitamin A on malignant tumors caused by sunburn was investigated. Our results showed that for people with skin cancers, serum vitamin A and β-cryptoxanthin may participate in the development of skin cancers and account for 5.89 and 2.14% effects apart from the influence of sun, respectively. And for people with non-skin cancers, serum zeaxanthin, β-cryptoxanthin, vitamin A, combined lutein, and *trans*-lycopene may participate in the development of their cancers, which account for 9.88, 4.79, 4.45, 3.11, and 1.32% effects apart from the influence of sun, respectively.

However, there were some limitations in our study. One is that this was a cross-sectional study design and cannot infer causal relationships. Our data from NHANES are based on self-reported outcomes, which cannot exclude recall bias. In addition, we focused only on the reaction after non-exposure and did not take sun protection behaviors into consideration, which may potentially affect the risks of cancers because there may be a possibility that if a person reacted severely to the sun, he or she may take sun protection or reduce the time of sun exposure. Additionally, the single measurement of serum carotenoids may not provide fully representative indications of carotenoid levels over a duration or the changes before and after sun exposure. Finally, because of the nature of this study and design, we cannot determine the optimal levels of carotenoids. Given the large sample size, the data provide valuable information regarding this cohort’s sunburn reaction in relation to cancer and carotenoid status.

In conclusion, serum carotenoids were associated with the severity of sunburn, and different carotenoids may play different in the context of sunlight exposure. Sunburn is related to not only in skin cancers but also non-skin cancers, and the more severe the sunburn reaction is, the higher the risk of cancer. Sunburn or sunlight may directly cause or promote cancers, but our results implied that some carotids may also take part in this process, partially impacting the effect of sunburn on skin and non-skin cancers. Overall, carotenoids may be important vitamins for skin protection and cancer prevention.

## Data availability statement

The raw data supporting the conclusions of this article will be made available by the authors, without undue reservation.

## Ethics statement

Ethical review and approval was not required for the study on human participants in accordance with the local legislation and institutional requirements. Written informed consent for participation was not required for this study in accordance with the national legislation and the institutional requirements. Written informed consent was not obtained from the individual(s) for the publication of any potentially identifiable images or data included in this article.

## Author contributions

All authors listed have made a substantial, direct, and intellectual contribution to the work, and approved it for publication.

## References

[1] Antille C Tran C Sorg O Carraux P Didierjean L Saurat J. Vitamin A exerts a photoprotective action in skin by absorbing ultraviolet B radiation. *J Invest Dermatol.* (2003) 121:1163–7. 10.1046/j.1523-1747.2003.12519.x 14708621

[2] Balic A Mokos M. Do we utilize our knowledge of the skin protective effects of carotenoids enough? *Antioxidants.* (2019) 8:259. 10.3390/antiox8080259 31370257 PMC6719967

[3] Baswan S Klosner A Weir C Salter-Venzon D Gellenbeck K Leverett J Role of ingestible carotenoids in skin protection: a review of clinical evidence. *Photodermatol Photoimmunol Photomed.* (2021) 37:490–504. 10.1111/phpp.12690 33955073

[4] Bouayed J Bohn T. Exogenous antioxidants–double-edged swords in cellular redox state: health beneficial effects at physiologic doses versus deleterious effects at high doses. *Oxid Med Cell Longev.* (2010) 3:228–37. 10.4161/oxim.3.4.12858 20972369 PMC2952083

[5] Caini S Gandini S Sera F Raimondi S Fargnoli M Boniol M Meta-analysis of risk factors for cutaneous melanoma according to anatomical site and clinico-pathological variant. *Eur J Cancer.* (2009) 45:3054–63. 10.1016/j.ejca.2009.05.009 19545997

[6] Catanzaro E Bishayee A Fimognari C. On a beam of light: photoprotective activities of the marine carotenoids astaxanthin and fucoxanthin in suppression of inflammation and cancer. *Mar Drugs.* (2020) 18:544. 10.3390/md18110544 33143013 PMC7692561

[7] Cenariu D Fischer-Fodor E Tigu A Bunea A Virag P Perde-Schrepler M Zeaxanthin-rich extract from superfood lycium barbarum selectively modulates the cellular adhesion and MAPK signaling in melanoma versus normal skin cells in vitro. *Molecules.* (2021) 26:333. 10.3390/molecules26020333 33440679 PMC7827977

[8] Cooperstone J Tober K Riedl K Teegarden M Cichon M Francis D Tomatoes protect against development of UV-induced keratinocyte carcinoma via metabolomic alterations. *Sci Rep.* (2017) 7:5106. 10.1038/s41598-017-05568-7 28698610 PMC5506060

[9] Eggersdorfer M Wyss A. Carotenoids in human nutrition and health. *Arch Biochem Biophys.* (2018) 652:18–26. 10.1016/j.abb.2018.06.001 29885291

[10] Centers for Disease Control and Prevention. *The National Health and Nutritional Examination Survey (NHANES).* (2022). Available online at: https://www.cdc.gov/nchs/nhanes/index.htm (accessed October 15, 2022).

[11] Grudzinski W Piet M Luchowski R Reszczynska E Welc R Paduch R Different molecular organization of two carotenoids, lutein and zeaxanthin, in human colon epithelial cells and colon adenocarcinoma cells. *Spectrochim Acta A Mol Biomol Spectrosc.* (2018) 188:57–63. 10.1016/j.saa.2017.06.041 28689079

[12] Ito N Seki S Ueda F. The protective role of astaxanthin for UV-induced skin deterioration in healthy people-A randomized, double-blind, placebo-controlled trial. *Nutrients.* (2018) 10:817. 10.3390/nu10070817 29941810 PMC6073124

[13] Kim J Lee J Oh J Chang H Sohn D Kwon O Dietary lutein plus zeaxanthin intake and DICER1 rs3742330 A > G polymorphism relative to colorectal cancer risk. *Sci Rep.* (2019) 9:3406. 10.1038/s41598-019-39747-5 30833603 PMC6399314

[14] Kim J Park M Li W Qureshi A Cho E. Association of vitamin A intake with cutaneous squamous cell carcinoma risk in the United States. *JAMA Dermatol.* (2019) 155:1260–8. 10.1001/jamadermatol.2019.1937 31365038 PMC6669777

[15] Kim J Lee J Choi I Kim Y Kwon O Kim H Dietary carotenoids intake and the risk of gastric cancer: a case-control study in Korea. *Nutrients.* (2018) 10:1031. 10.3390/nu10081031 30087311 PMC6115955

[16] Kopcke W Krutmann J. Protection from sunburn with beta-Carotene–a meta-analysis. *Photochem Photobiol.* (2008) 84:284–8. 10.1111/j.1751-1097.2007.00253.x 18086246

[17] Le Marchand L Saltzman B Hankin J Wilkens L Franke A Morris S Sun exposure, diet, and melanoma in Hawaii Caucasians. *Am J Epidemiol.* (2006) 164:232–45. 10.1093/aje/kwj115 16524953

[18] Meinke M Friedrich A Tscherch K Haag S Darvin M Vollert H Influence of dietary carotenoids on radical scavenging capacity of the skin and skin lipids. *Eur J Pharm Biopharm.* (2013) 84:365–73. 10.1016/j.ejpb.2012.11.012 23246796

[19] Nakamura H Takada K. Reactive oxygen species in cancer: current findings and future directions. *Cancer Sci.* (2021) 112:3945–52. 10.1111/cas.15068 34286881 PMC8486193

[20] Naldi L Gallus S Tavani A Imberti G La Vecchia C. Oncology study group of the Italian group for epidemiologic research in D. risk of melanoma and vitamin A, coffee and alcohol: a case-control study from Italy. *Eur J Cancer Prev.* (2004) 13:503–8. 10.1097/00008469-200412000-00007 15548944

[21] Nash S Till C Song X Lucia M Parnes H Thompson I Jr. Serum retinol and carotenoid concentrations and prostate cancer risk: results from the prostate cancer prevention trial. *Cancer Epidemiol Biomarkers Prev.* (2015) 24:1507–15. 10.1158/1055-9965.EPI-15-0394 26269564 PMC4592455

[22] Nishino A Sugimoto K Sambe H Ichihara T Takaha T Kuriki T. Effects of dietary paprika xanthophylls on ultraviolet light-induced skin damage: a double-blind placebo-controlled study. *J Oleo Sci.* (2018) 67:863–9. 10.5650/jos.ess17265 29877227

[23] Ruhl R. Effects of dietary retinoids and carotenoids on immune development. *Proc Nutr Soc.* (2007) 66:458–69. 10.1017/S002966510600509X 17637099

[24] Sheng Y Luo Y Liu S Xu W Zhang Y Zhang T Zeaxanthin induces apoptosis via ROS-regulated MAPK and AKT signaling pathway in human gastric cancer cells. *Onco Targets Ther.* (2020) 13:10995–1006. 10.2147/OTT.S272514 33149614 PMC7605660

[25] Sies H Stahl W. Carotenoids and UV protection. *Photochem Photobiol Sci.* (2004) 3:749–52. 10.1039/b316082c 15295630

[26] Stahl W Sies H. Photoprotection by dietary carotenoids: concept, mechanisms, evidence and future development. *Mol Nutr Food Res.* (2012) 56:287–95. 10.1002/mnfr.201100232 21953695

[27] Xavier A Perez-Galvez A. Carotenoids as a source of antioxidants in the diet. *Subcell Biochem.* (2016) 79:359–75. 10.1007/978-3-319-39126-7_14 27485230

[28] Xie L Song Y Lin T Guo H Wang B Tang G Association of plasma retinol levels with incident cancer risk in Chinese hypertensive adults: a nested case-control study. *Br J Nutr.* (2019) 122:293–300. 10.1017/S000711451900120X 31352906

